# Nutrition in the Prevention of Breast Cancer: A Middle Eastern Perspective

**DOI:** 10.3389/fpubh.2019.00316

**Published:** 2019-11-08

**Authors:** Farah Naja, Lara Nasreddine, Sara Awada, Raeda El Sayed Ahmad, Nahla Hwalla

**Affiliations:** Department of Nutrition and Food Science, Faculty of Agricultural and Food Sciences, American University of Beirut, Beirut, Lebanon

**Keywords:** breast cancer, diet, risk factors, prevention, obesity, Middle East and North Africa region

## Abstract

This paper reviews the escalating burden of breast cancer (BC) in the Middle East (ME) and the prevalence of modifiable risk factors and underscores opportunities to promote the prevention of the disease. Similar to more developed countries, BC is the most frequent cancer among women in countries of the ME, accounting for one-third of total cancer cases and 24% of total cancer deaths. Average age at BC diagnosis appears to be a decade earlier in Middle Eastern countries compared to the Western countries, and its incidence is predicted to further increase. Although incidence rates of BC are still lower in Middle Eastern countries than Western ones, mortality rates are similar and at times even higher. It is estimated that 30% of BC cases are due to environmental and lifestyle factors, such as obesity and diet and hence can be preventable. The ME suffers from surging rates of obesity, with eight of its countries ranking among the highest worldwide in obesity prevalence among adults aged 18 and above. ME countries with the highest prevalence of obesity that are among the top 20 worldwide include United Arab Emirates (UAE), Lebanon, Egypt, Libya, Qatar, Saudi Arabia, Jordan, and Kuwait with rates ranging from 30% in UAE to 37% in Kuwait. In parallel, studies in the ME have consistently showed a shift in dietary intake whereby traditional diets, rich in fruits and vegetables, are progressively eroding and being replaced by westernized diets high in energy and fat. Accumulating evidence is reporting convincing association between consumption of such westernized diets and higher BC risk. Addressing these risk factors and studying their association with BC in terms of their nature and magnitude in Middle Eastern countries could provide the basis for intervention strategies to lower the risk and alleviate the burden of BC in these countries.

## Introduction

Globally, the most common cancer among women is breast cancer (BC), representing about 25% of all cancers. BC incidence rates vary widely across the world, from 25 per 100,000 in Middle Africa and Eastern Asia to 92 per 100,000 in Western Europe. Incident cases are estimated to increase worldwide by 46.5% by the year 2040 (1).

In the Middle East (ME), the age-standardized incidence rate (ASR) of BC is 45.3 per 100,000 females and is substantially increasing with predictions to reach Western levels (1). According to the World Health Organization (WHO) estimates, BC rates across the ME are expected to double between 2012 and 2030, which is the highest relative increase of any region globally (2). Although ASR of BC per 100,000 in the ME is lower than that of Europe and the US (45.3 vs. 80.1 and 84.8, respectively), it has a similar mortality rate compared to these countries (13.6 vs. 12.6 and 14.1, respectively) (1). It is noteworthy that, in Middle Eastern countries, the incidence of BC occurs in women at an average age of diagnosis of <50 years, which is around 10 years before it appears in industrialized countries (3, 4). As shown in Table 1, limited available data from the Middle East and North Africa (MENA) region shows an increase in ASR of BC per 100,000 females. For example, in Lebanon, over a period of 12 years (1996–2008), ASR of BC has more than quadrupled, from 20 to 95.7. Also, in Jordan between the years 1982 and 2008, ASR increased by more than 6-fold, from 7.6 to 50.4. This illustrates the increasing trajectory of BC in this region (5, 6). It should be cautioned that the reported increases in the incidence of BC in the ME over the last decade may be attributed, in part, to the increase in number of cancer registries, as well as to the wide adoption of mammographic screening programs, an effort supported by several awareness campaigns since 2004 (1, 7, 8).

**Table 1:** Breast cancer age-standardized incidence rates ASR (World Population) per 100,000 females in the MENA countries over time.

**Breast cancer**	**Lebanon**	**Jordan**	**Saudi Arabia**	**Oman**	**Algeria**	**Morocco**	Egypt^*^	**Israeli Jewish**	**Israeli Arabs**
ASR per 100,000	20 (1996)	7.6 (1982)	–	–	–	–	–	–	–
	69 (2003)	32.8 (1997)	–	–	–	–	–	–	–
	71 (2004)	41.4 (2005)	15.4 (2004)	21.9 (2006)	23.5 (2002)	35 (2004)	49.6 (2001)	–	42.6 (2002)
	95.7 (2008)	50.4 (2008)	22.7 (2008)	21.6 (2008)	65.1 (2007)	36.4 (2005–2007)	Ranges from 35.7 to 63.9 (2009)	94.3 (2008)	56.2 (2008)

*Adapted from Lakkis et al. (5)*.

*Lebanese Ministry of Public Health-National Cancer Registry, May 2009*.

Abulkhair et al. (6).

* *ASRs of Egypt (Aswan), Egypt (Damietta), and Egypt (Minia) are 63.9, 41.4, and 35.7, respectively*.

Prevention strategies have been assessed globally showing that a minimum of 1.3 million cancer deaths and 30% of all cancer cases can be avoided yearly if healthy living and adequate working environments were sustained (9). The WCRF/AICR specified a few environmental and lifestyle factors that showed compelling evidence for their implication in the onset of BC, namely, smoking, diet, obesity, alcohol, sun exposure, physical activity, stress, pollution, and infections (10). Among these factors, obesity and the shift in dietary intake patterns are perceived as important modifiable risk factors of BC. In fact, the increase in BC incidence in Middle Eastern countries was concomitant with the escalating rates of obesity and the shift in dietary patterns (1, 11).

Thus, exploring the underlying factors that are associated with the increased risk of BC in the ME provides a foundation for intervention strategies to mitigate the risk of this cancer in the region. This mini-review examines the escalating burden of BC in the ME and the prevalence of modifiable risk factors and underscores the opportunities to promote prevention of this disease. A total of 71 articles for this minireview were collected from the two search engines PubMed and Google Scholar. Publications in English were selected and search terms included breast cancer, diet/nutrition, and breast cancer, risk factors of breast cancer, prevention of breast cancer, obesity and breast cancer, and breast cancer in the Middle East and North Africa (MENA) region.

## Obesity and BC

Among the modifiable risk factors strongly associated with BC is weight gain. In a meta-analysis by Cheraghi et al., the effect of obesity and overweight on BC in pre- and post-menopausal periods was examined through 15 cohort studies and 35 case–control studies (12). The results revealed that the BC's incidence increased by 14% among overweight and obese women in the post-menopausal stage whereas body mass index (BMI) did not have a significant effect on BC's incidence during the premenopausal stage (12). Similar to these findings, another meta-analysis summarized the results of 9 cohort studies and 22 case–control studies and showed that with every 5 units increase in BMI, BC's risk among postmenopausal women increases by 33% and decreases by 10% among premenopausal women (13). Building on available evidence, the continuous update project (CUP) panel graded the evidence regarding the association between BC and increased body fatness and weight gain as convincing for post-menopausal women whereas the protective effect of body fat against BC in pre-menopausal was graded as probable (14). It is therefore suggested that the risk of BC could be mediated by the menopausal state. More recent data on the distinctive effect of body fat on BC revealed a 12% increase in BC risk among overweight postmenopausal women, which further increased to 25% in obese postmenopausal women (15). High levels of body fat were also associated with an increase in BC risk among postmenopausal women with normal BMI (16, 17).

In several MENA countries, the prevalence of overweight and obesity are at alarmingly high levels where 66–75% of the adult population in the Gulf countries are estimated to be overweight and obese (1, 18). Compared to worldwide figures, eight Middle Eastern countries are among the top 20 countries with the highest prevalence of obesity. These include United Arab Emirates (UAE), Lebanon, Egypt, Libya, Qatar, Saudi Arabia, Jordan, and Kuwait with rates ranging from 30% in UAE to 37% in Kuwait, the latter being among the top 10 countries with highest obesity rates worldwide (19). More specifically, the MENA countries have one of the highest rates of female obesity prevalence on earth and have experienced more rapid increase in incidence of obesity than the developed countries between the years 1990 and 2016 (20). The percent increase in obesity in males and females in 26 years were 170 and 81% in MENA countries as compared to 122 and 75.5%, respectively, in the world. Examining the percent contribution of obesity to BC, data show that the percentages of post-menopausal BC cases attributable to excess BMI among females ranges between 15.2% in Lebanon and 18.5% in Kuwait (Table 2) (1).

**Table 2:** Percentages of all post-menopausal breast cancer cases among females worldwide in 2012 attributable to excess body mass index, by country from highest to lowest.

**Rank**	**Country**	**Percentage (%)**
1	Samoa	20.2
2	Kuwait	18.5
3	Jordan	18.1
4	Saudi Arabia	17.3
5	United Arab Emirates	17.3
6	Libya	17.1
7	West Bank and Gaza	17.1
8	Puerto Rico	17.1
9	Egypt	16.9
10	Syria	16.4
11	South Africa	16.3
12	Turkey	16.2
13	Bahamas	16.1
14	Qatar	15.6
15	Fiji	15.4
16	Barbados	15.3
17	Lebanon	15.2
18	Belize	15.2

*Adapted from Bray et al. (1), IARC World Health Organization, http://gco.iarc.fr/causes/obesity/tools-map*.

Several mechanisms were reported, in the literature, attempting to explain the relationship between obesity and BC. It was proposed that the insulin resistance of obesity is linked to metabolic abnormalities that may lead to a decrease in insulin-like growth factor binding protein 1 and insulin-like growth factor binding protein 2, which, in turn, increases the bioavailability of insulin-like growth factor 1 hence, promoting cellular proliferation and inhibiting apoptosis. These events could promote tumorigenesis (21).

Other possible mechanisms supporting the relationship between body fatness and BC are related to increased adiposity. The adipose tissue is an active metabolic organ, with excess adiposity associated with endocrine and metabolic characteristics, altered adipokines (higher leptin and lower adiponectin levels), inflammation, and higher estrogen levels, all of which may inhibit apoptosis and promote tumorigenesis. Many of these factors have been studied and shown to have a link with increased risk of BC, notably in postmenopausal women (22).

## Genetic Predisposition and BC

BRCA1/2 mutation is a known hereditary risk factor for BC, whereby in Western populations, this mutation confers a lifetime risk of BC of up to 80%, with up to 40% of carriers developing BC by the age of 50 (23). A systematic review of studies examining BRCA1 mutation in the MENA concluded that this mutation is rather frequent in this part of the world and that each region within the MENA appears to have unique mutations (7, 24–31). The authors of this systematic review recommended the development of a mutation database, by each region, for BC screening. National data on BRCA1 mutations may be targeted for this screening to get the best estimation of this cancer-promoting mutation (32). For example, in 2019, and in line with the latter recommendation, the BRCA1 c.131G mutation was considered a founder mutation in the Lebanese population as it was detected among 23% of individuals diagnosed with BRCA mutation, and in Turkey, the positivity prevalence of BRCA1/2 mutation was 19% in high-risk BC patients (31, 33).

## Dietary Patterns and BC

Diet quality has been reported as another modifiable BC risk factor. It is estimated that almost one-third of the BC cases can be prevented through dietary modifications (14, 18, 34, 35).

Meta-analysis studies on dietary patterns and BC revealed that, of three different patterns studied in both developed and developing countries, the prudent diet, which is a diet rich in fruits, vegetables, legumes, poultry, fish, whole grains, and low-fat dairy, had a protective effect on BC with an 11% decreased risk. Alternatively, the Western/unhealthy dietary pattern and the drinker dietary pattern had detrimental effects on BC as they were respectively associated with a 9 and 21% increased risk of BC (34). More specifically, unhealthy dietary patterns, such as those high in sugar, trans fats, refined carbohydrates, and alcohol along with low intake of fibers, antioxidants, and omega 3 fatty acids were shown to increase the risk of BC (15). Similarly, a systematic review of 17 case–control studies identified that dietary patterns that include vegetables, fruits, lean protein, grains, and legumes may reduce the risk of BC, whereas dietary patterns that include high saturated fats, fried foods, sugars, refined grains, and processed meats may increase the risk of BC (36). Also, a 10% increase in ultra-processed foods, such as packaged goods, sugary cereals, and ready meals was found to increase the risk of BC by 12% (37).

Over the last decade, the MENA region was reported as undergoing a shift in the dietary patterns from the traditional healthy Mediterranean type diet to a more westernized diet rich in energy and fat. The diet is becoming energy-dense, sweet, high in fat and processed foods, and low in fiber, cereals, fruits, and vegetables (38). The results of a case–control study from the Kingdom of Saudi Arabia (KSA) suggested a positive association between fats intake, protein, and calories and BC risk (39). This could be associated with the increase in BC incidence among women in these countries. In light of the protective association between the traditional diet and BC risk, increased efforts are needed to promote shifting the dietary patterns to the traditional healthy Mediterranean diet of this region (18).

## Alcohol and BC

Alcohol is considered as a promoting factor of human carcinogenesis. It is a well-established modifiable risk factor for BC, being significantly associated with post-menopausal BC and accounting for 5% of worldwide BC deaths (10, 15, 40).

CUP identified four published pooled analyses on the risk of pre- and post-menopausal BC and consumption of alcohol. The results showed that the evidence was consistent, and the increased BC risk remained significant in all studies. In this context, CUP also identified 22 studies that were included in the dose-response meta-analysis, whose results showed a 9% increased risk of BC in the post-menopausal state per 10-g (equivalent to 330 ml of beer and 100 ml of wine) increase in alcohol consumed per day. Hence, CUP graded the evidence for the association between consumption of alcoholic drinks and BC as convincing in postmenopausal women and as probable in pre-menopausal ones CUP, 2018 (14).

Research about the association between alcohol consumption and BC in the ME has been hampered by societal and religious traditions. It was reported that the consumption of alcohol among Middle Eastern women is not viewed as a major problem due to low consumption, and hence it may not be substantially contributing to the rise of BC incidence and deaths in these countries (18).

Many possible mechanisms speculating on the association between alcohol and BC were reported, mainly suggesting that enzymatic degradation of alcohol is linked with a change in the proportions of the two forms of the coenzyme nicotinamide adenine dinucleotide (NAD). The accumulation of its reduced form, nicotinamide-adenine dinucleotide- hydrogen (NADH), means that the breakdown of estradiol to estrone is less favored and estradiol accumulates, hence increasing the rate of aromatization of testosterone to estradiol. The binding of estrogens to its nuclear receptor (ERα) initiates a complex intracellular signal sequence, finally stimulating cell proliferation and cancer (41).

## Red and Processed Meat and BC

High intake of red and processed meats was reported to be associated with increased risk of BC. The Women's cohort study in the UK, the NIH-AARP Diet and Health Study, and the Nashville Breast Health study showed that there was an increased risk of BC in both pre- and post-menopausal women who had high consumption of red meat (15). Another prospective cohort study showed that increased consumption of red and processed meat among adolescent females was linked to increased risk of premenopausal BC (18). A meta-analysis of 14 prospective studies on red meats and 12 prospective studies on processed meats indicated that there is a 10% increased risk of BC due to high intake of red meats (120 g/day) and an 8% increased risk due to high intake of processed meats (50 g/day) (42). A case–control study from Iran suggests that consuming red meat is associated with increased risk of BC (43). Previous reports by the WCRF/AICR 2007 stated that the safe intake level of cooked red meat should not exceed 500 g/week (equivalent to 71.4 g/day) and the intake of processed meats should be avoided. Middle Eastern countries have high intakes of red and processed meats, and most of these countries surpass the recommended levels where, for example, the consumption of processed meats in UAE, Algeria, Kuwait, and Lebanon was estimated to be 47, 17.5, 42, and 32 g/week, respectively; as for red meats, it was reported to be 700, 707, 700, and 400 g/week, respectively (44).

CUP graded the evidence of the link between high intake of red and processed meats and increased risk of BC as limited in both pre- and post-menopausal women, which calls for further studies to understand these potential associations. Among the possible reported explanations for the link between meat and BC are the high-fat intake associated with consuming fatty meat, and polycyclic aromatic hydrocarbons and heterocyclic amines formed during meat cooking, which are considered human carcinogens (18, 45, 46).

## Fruits, Vegetables, and BC

Several studies documented the high intake of fruits and vegetables as protective against BC in women (47, 48). A meta-analysis of 14 cohort studies and 1 case–control study indicated that a high intake of fruits and vegetables combined (>400 g/day for fruits and >300 g/day for vegetables), but not vegetables alone, is associated with an 11% decrease in BC risk (49). Similarly, a meta-analysis of 11 case–control studies and 2 cohort studies showed that a high intake of cruciferous vegetables is significantly linked to a 15% reduction in BC risk. In this meta-analysis, cruciferous vegetables were referred to arugula, broccoli, Brussels sprouts, bok choy, cabbage, canola, cauliflower, collard greens, daikon, horseradish, kale, kohlrabi, mustard, radish, rutabaga, wasabi, and watercress (50). Based on these studies, it was suggested that the intake of cruciferous vegetables and fruits have a protective effect on BC in pre- and post-menopausal women. Studies on food consumption in many countries of the ME and in 22 Arab countries showed a low intake of fruits and vegetables among adults in this region, which is less than the recommended daily intake (above 400 g) among females of all age groups (51). The lowest intakes of fruits and vegetables were seen in Libya (fruits 60.4 g/day and vegetables 134 g/day), Algeria, Yemen, Iran, and Iraq (less than the optimal intake, which is 400 ± 30 g/day) (18, 44).

Several mechanisms may explain the protective effect of fruits and vegetables against BC. Fruits and vegetables are good sources of fiber, which may bind to estrogens, inhibiting the process of enterohepatic reabsorption of estrogen (52). Fruits and vegetables are also very good sources of various antioxidants including glucosinolates, carotenoids, indoles, and isothiocyanates, which can help prevent BC by inducing detoxifying enzymes and decreasing oxidative stress and inflammation (49, 53). More studies in the MENA region are needed to investigate the link between fruits and vegetables' intakes and BC risk in women.

## Fish, Marine n-3 polyunsaturated fatty acids, and BC

Fish rich in omega-3 polyunsaturated fatty acids (n-3 PUFA) were reported as being associated with a decreased risk of BC among females. A meta-analysis of five cohort and six prospective case–control studies indicated that there was a 6% reduction in BC risk in the study populations from the United States, Europe, and Asia following a 1/10 increment of n-3/n-6 ratio in the diet (54). Similarly, a meta-analysis of 21 prospective cohort studies showed that a higher intake of dietary marine n-3 PUFA was associated with a lower risk of BC. The risk was decreased by 5% following 0.1 g/day increase in the intake of dietary marine n-3 PUFA (55).

Studies investigating food consumption patterns in countries of the ME have reported low intakes of marine n-3 fats (less than the optimal recommended level by the Academy of Nutrition and Dietetics of 500 mg/day of EPA and DHA of which at least 220 mg should consist of EPA). The lowest intakes were seen in Lebanon Palestine, Syria, Algeria, Iraq, Qatar, Jordan, and Oman (44).

Reports proposing mechanisms by which n-3 PUFA could influence BC risk suggested eicosanoids, n-3 PUFA metabolites, as modulators of cellular processes either by interacting with receptors or by altering signaling pathways. This may result in downregulating the inflammatory cascade, enhancing fatty acid (FA) degradation in association with lowering FA synthesis, and lowering the expression of markers ultimately increasing cell death (56).

## Fiber and BC

Diets rich in fiber were reported to be linked to a reduced BC risk. A meta-analysis of 16 prospective studies indicated that there is an inverse association between the intake of dietary fiber and risk of BC (5% reduction) (57).

Middle Eastern populations, especially Turkey, Egypt, Kuwait, Jordan, Yemen, and UAE, have low intakes of whole grains less than the optimal level of 50 g/day, which constitutes the main source of dietary fibers (44, 58). Other studies targeting specific types of fiber and the BC risk among pre- and post-menopausal women were reported as needed to clarify the mechanisms behind the positive effect of dietary fiber on BC (59). Several mechanisms of action of fiber in protection from BC were proposed in the literature; one mechanism is related to decreasing circulating estrogen levels and increasing fecal excretion of estrogen; hence, the binding of estrogen to its nuclear receptor ERα is hindered, and accordingly, cell multiplication is decreased (57). Another mechanism is the binding of fibers to bile acids, which are suggested to advance cell proliferation, thus allowing decreased chance for mutations and decreasing cancer risk (60). Fermentation of fibers produces butyrate, a short-chain fatty acid, which has been shown to have antineoplastic effects (61).

## Carbohydrates and BC

The association between carbohydrates and BC is unclear. A meta-analysis of 10 prospective cohort studies showed that high dietary glycemic index (GI) is significantly associated with an 8% increased risk of BC, and high dietary glycemic load (GL) is associated with a 3% increased risk of BC (62). Given the limited number of eligible studies to support the association of GI and BC in all countries, including Middle Eastern ones, more studies are needed to examine this association. Nevertheless, reducing the intake of high GI foods, notably refined carbohydrates, in the general population may perhaps offer a benefit in preventing BC (62). Speculating on possible mechanisms regarding the relationship between carbohydrates and BC, the literature suggests that high insulinemia, in response to high glycemic index diets, may inhibit apoptosis and synthesis of IGF1-1 binding proteins 1 and 2, which promote cellular multiplication (63).

## Vitamin D and BC

A meta-analysis of two randomized clinical trials and one prospective cohort showed that women with 25(OH) D concentration of ≥60 ng/ml had an 80% lower BC incidence rate than women with concentration <20 ng/ml (64). Also, another meta-analysis of 14 case–control studies indicated that serum 25(OH)D concentration was inversely and significantly associated with 16% decreased BC risk (65). Similarly, a case–control study from KSA in the ME showed an inverse association between serum 25(OH) D, the active form of vitamin D, and the risk of BC in Saudi women (66). However, several other studies have shown no association between dietary and supplemental vitamin D and BC (67–69).

The level of 25(OH) D is considered deficient if it is <25 nmol/L, insufficient if it is between 25 and 49 nmol/L (<20 ng/ml), and inadequate if it is between 50 and 74 nmol/L (70). Studies in the ME showed that the highest prevalence of vitamin D deficiency was found among women (6). For instance, 81% of adolescent girls in Saudi Arabia and 62% in Qatar have vitamin D deficiency (<12 ng/ml). As for adult women, 37% in Jordan and 51% in Iran have vitamin D insufficiency (<20 ng/ml) (71). Altogether, the evidence that 25(OH) D decreases the risk of BC is labeled by CUP as probable (14).

The mechanism by which vitamin D can affect BC has been speculated in the literature, stating that the biologically active form of vitamin D binds to the vitamin D receptor in normal breast epithelium and this complex regulates the cell cycle, promotes differentiation, increases cell-to-cell adhesion, protects cells from DNA damage, regulates cytokines, activates immune cells, and suppresses inflammation, thus reducing malignant transformations (72).

## Conclusion

In summary, there is sufficient research to suggest an association between obesity and nutrition with BC globally and regionally. In the ME, the rise in the rates of new BC cases, especially among younger women, coupled to the alarming levels of obesity and the shift in dietary patterns toward westernized diet call for action in all countries and at all levels of the society. Policies, strategies, and public health efforts to reduce obesity and promote a healthy lifestyle with emphasis on the prudent diet are needed. It remains important to note that such public health interventions are hampered by the scarcity of research and data that provide a local, context-specific, and culturally adaptable evidence base. The evidence presented in this paper points toward ethnic and context-specific associations between BC and the reported risk factors. This may trigger systematic and well-designed studies in the ME to affirm all these associations, assess the genetic predisposition to BC, and provide data for region-specific evidence-based recommendations for the prevention of BC.

## Strengths and Limitations

A few limitations ought to be considered when interpreting the findings of this minireview. First, the associations of BC with obesity and nutrition are complex, especially that BC is a disease with a multifactorial and complex etiology of genetics as well as environmental factors. Second, there is a paucity of region-specific studies investigating the association between diet, lifestyle, and BC; hence, most associations of risk factors with BC were conducted in Western countries. Moreover, despite our efforts to include all relevant meta-analyses on BC in MENA, the potential of selection bias could not be ruled out.

## Author Contributions

NH conceptualized the content of the chapter and acted as lead author of the manuscript. FN and LN provided critical review of the manuscript and participated in the write up. SA conducted the literature search and contributed to the write up of the chapter. RE contributed to the editing and the write up of the chapter.

### Conflict of Interest

The authors declare that the research was conducted in the absence of any commercial or financial relationships that could be construed as a potential conflict of interest.
